# A Cross-Sectional Study on Nutritional and Morbidity Status of Children Attending Anganwadi Centers in Andhra Pradesh, India

**DOI:** 10.7759/cureus.21794

**Published:** 2022-01-31

**Authors:** Archana Carolin, Surya Balakrishnan, Renuka Senthil

**Affiliations:** 1 Community Medicine, Panimalar Medical College Hospital and Research Institute, Chennai, IND

**Keywords:** anganwadi centre, anthropometry, icds, anemia, malnutrition

## Abstract

Background

The health status of children is considered a very important and vital factor for building the future of a growing nation. So providing proper nutrition to the child is very important to enhance the growth of the child.

Objectives

The present study is intended to assess the nutritional status and morbidity pattern of children attending Anganwadi centers.

Materials and methods

This community-based cross-sectional study was conducted in children in the age group of 3-6 years attending the Anganwadi centers. By using the simple random sampling technique 13 Anganwadi centers were selected and a sample of 381 children was selected as the study population. Data was entered and analyzed using SPSS version 17 (Chicago: SPSS Inc). Ethical clearance was obtained from the institutional ethical committee (ECR/460/Inst/AP/2013/RR_19).

Results

The overall prevalence of underweight, stunting, and wasting was 172 (45.2%), 243 (63.8%), and 79 (20.7%), respectively, according to the WHO-recommended classification. It was observed that a high prevalence of morbidity was of anemia, 125 (32.08%), followed by upper respiratory tract infection, 78 (20.48%).

Conclusion

The present study shows that there are still many children who are malnourished and suffering from anemia in our country, even after 46 years of Integrated Child Development Services (ICDS). As malnutrition is a complex and multi-dimensional issue, comprehensive studies regarding causative, aggravating, and associated factors leading to malnutrition are required to be studied, to know the problem in-depth, and formulate better health policies.

## Introduction

Every country in the world is affected by one or more forms of malnutrition. Combating malnutrition in all its forms is one of the greatest global health challenges. Globally in 2020, 149 million children under 5 were estimated to be stunted (too short for age), 45 million were estimated to be wasted (too thin for height), and 38.9 million were overweight or obese. Around 45% of deaths among children under 5 years of age are linked to undernutrition. Malnourished children, particularly those with severe acute malnutrition, have a higher risk of death from common childhood illnesses such as diarrhea, pneumonia, and malaria. Nutrition-related factors contribute to about 45% of deaths in children under 5 years of age [1].

The Integrated Child Development Services (ICDS) program was started in 1975 in pursuance of the National Policy for Children. There is a strong nutrition component in this program in the form of supplementary nutrition, vitamin A prophylaxis, iron and folic acid distribution. The beneficiaries are preschool children below 6 years, adolescent girls 11 to 18 years, and pregnant and lactating mothers. The workers at the village level who deliver the services are called Anganwadi workers. Each Anganwadi unit covers a population of about 400 to 800 and mini Anganwadi center about 150 to 400. The work of Anganwadi centers is supervised by Mukhyasevikas. Field supervision is done by the Child Development Project Officer (CDPO) [2].

Children from rural areas, slums and urban poor families, scheduled castes, tribal communities, and other disadvantaged populations suffer from multiple deprivations related to poverty, malnutrition, access to quality health services, child marriage, poor school attendance, low learning outcomes, lack of sanitation facilities, hygiene, and access to improved water. Children from rural areas are more likely to die before completing age five than those living in urban areas [3].

In the present study, an attempt was made to explore the nutritional status and morbidity pattern of children attending Anganwadi centers, especially 3-6-year-old beneficiaries belonging to the Rural Field Practice Area, Venkatachalam, Nellore district, Andhra Pradesh (India). 

## Materials and methods

This community-based cross-sectional study was conducted in children in the age group of 3-6 years attending the Anganwadi centers of Venkatachalam, Rural Field Practice Area of Department of Community Medicine, Narayana Medical College, Nellore, Andhra Pradesh, for a period of one year from June 2013 to May 2014. There is a total of 25 Anganwadi centers present in the rural field practice area. By using a simple random sampling technique, 13 Anganwadi centers were selected and included in the study. According to the National Family Health Survey - 3 (NFHS-3), the prevalence of undernutrition was 40% [4]. Based on that, the sample size was calculated by using the formula n=3.84pq/d2 with 5% of absolute precision. The sample size calculated was 368. The total number of children enrolled in 13 Anganwadi centers was 633, out of which only 381 children were present during the visit. Since that is is closer to the sample size calculated, all the children were included in the study.

The data was collected by interviewing the Anganwadi teacher by using a pre-designed questionnaire, which was validated after a pilot study. Additional information was gathered using individual health records. A general physical examination was done. Anthropometric measurements were done using Salter’s weighing scale for weight assessment and measuring tape for height measurement. The WHO Child Growth Standards, 2006 [5] reference data was used for that particular age and sex to get height for age (stunting), weight for age (underweight), and weight for height (wasting).

Data thus obtained was coded and entered into Microsoft Excel worksheet (Microsoft Corporation, Redmond, USA) and it was analyzed using SPSS version 17 (SPSS Inc., Chicago, USA). All the variables were presented as frequencies and percentages, and Chi-square tests were applied to assess the association of various variables studied in the research. P-value <0.05 was considered to be statistically significant.

Ethical considerations

Ethical clearance was taken from the Institutional Ethics Committee of Narayana Medical College, Nellore (ECR/460/Inst/AP/2013/RR_19), and the permission of the Child Development Project Officer (CDPO), Nellore District was taken before starting the study. Informed consent was obtained from the Anganwadi teacher and assent from the parents.

## Results

Out of the 381 children who were involved in the study, the majority were in the age group of 48-59 months - 141 (37%); female children with 196 (51.4%) outnumbered the male children; 348 (91.3%) children belonged to the Hindu religion. All the study participants were fully immunized (Table 1).

**Table 1:** Demographic characteristics of children attending Anganwadi centers

Demographic characteristics		Number n=381	Percentage (%)
Age in months	36-47	110	28.9
	48-59	141	37
	60-72	130	34.1
Gender	Male	185	48.6
	Female	196	51.4
Religion	Hindu	348	91.3
	Christian	009	2.4
	Muslim	024	6.3
Immunization status	Yes	381	100
	No	000	0

Based on the nutritional status according to WHO Growth Standards Reference (2006), the prevalence of underweight, stunting, and wasting was 172 (45.2%), 243 (63.8%), and 79 (20.7%), respectively. According to the measurement of mid-arm circumference, the prevalence of protein-energy malnutrition was found to be 17 (4.5%) (Table 2).

**Table 2:** Nutritional status of children attending Anganwadi centers PEM: protein-energy malnutrition

Nutritional status		Number (n=381)	Percentage (%)
Weight for age (underweight)	Normal	209	54.9
	Moderate	94	24.7
	severe	78	20.5
Height for age (stunting)	Normal	138	36.2
	Moderate	129	33.9
	severe	114	29.9
Weight for height (wasting)	Normal	302	79.3
	Moderate	52	13.6
	severe	27	7.1
Mid-arm circumference	Normal	364	95.5
	Mild to moderate PEM	17	4.5
	Severe PEM	0	0

The association between the gender and the nutritional status of the children - underweight, stunting, and wasting - was found to be statistically not significant with the p-value of 0.0793, 0.8324, and 0.5043, respectively (Table 3).

**Table 3:** Association of gender with nutritional status

Variable		Gender				
Nutritional Status		Male	Female	Total	Chi-square	P value
		N (%)	N (%)			
Underweight	Yes	75(43.6)	97(56.4)	172	3.0779	0.0793
	No	110(52.6)	99(47.4)	209		
Stunting	Yes	117(48.1)	126(51.9)	243	0.0448	0.8324
	No	68(49.3)	70(50.7)	138		
Wasting	Yes	41(51.9)	38(48.1)	79	0.4457	0.5043
	No	144(47.7)	158(52.3)	302		

The association between the age group and the stunting was found to be statistically significant with a p-value of 0.000132 (Table 4).

**Table 4:** Association of age group with nutritional status

variable		Age in Months					
Nutritional Status		36-47	48-59	60-72	Total	Chi-square	P value
		N (%)	N (%)	N (%)			
Underweight	Yes	55(32)	61(35.5)	56(32.5)	172	1.4733	0.4787
	No	55(26.3)	80(38.3)	74(35.4)	209		
Stunting	Yes	87(35.8)	87(35.8)	69(28.4)	243	17.87	0.000132
	No	23(16.7)	54(39.1)	61(44.2)	138		
Wasting	Yes	19(24.1)	28(35.4)	32(40.5)	79	2.059	0.3571
	No	91(30.1)	113(37.4)	98(32.5)	302		

**Figure 1:**
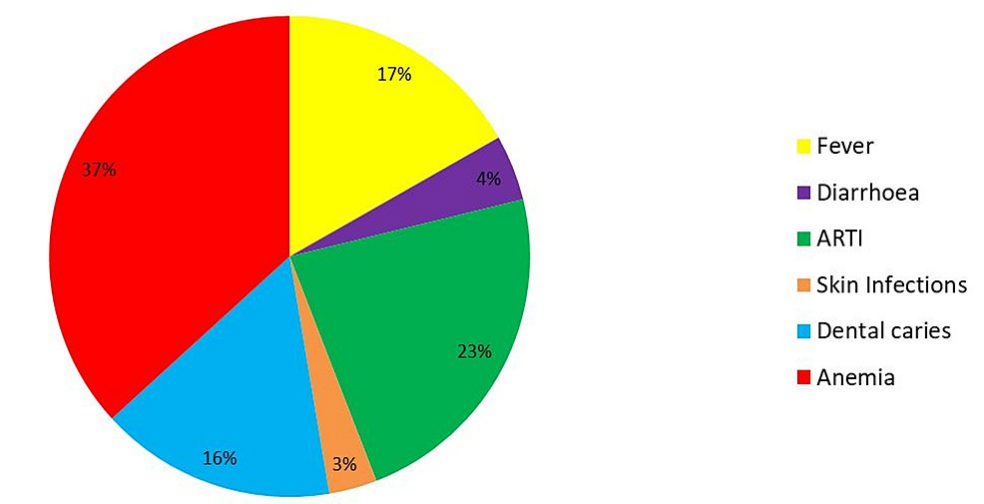
Morbidity status of the children attending Anganwadi center ARTI: acute respiratory tract infection

It was observed that the highest prevalence of morbidity was of anemia, 125 (32.80%), followed by upper respiratory tract infection, 78 (20.48%) (Figure 1).

## Discussion

In the present study comprising of 381 children, the majority of the children belonged to the 48-59 months age group - 141 (37%).

A study conducted by Shreyaswi et al. on the prevalence and risk factors of undernutrition among under-five children in a rural community found that out of 133 children surveyed, the majority of the children (43.6%) belong to the 4-5 year age group [6]. This study is similar to our study.

A study was done by Kumari and Thomas to assess the health profile of children (0-6 years) in ICDS vs. non-ICDS urban slums of Hyderabad found that out of 1200 children from ICDS-covered slum, the majority (23%) belonged to 5-6 years age group [7]. This is in contrast to our study's findings.

The study by Ukey and Chitre assessed the morbidity profile of pre-school children in an urban slum area of Visakhapatnam and found that out of 353 children, the majority (30.59%) belonged to the 4-5 years age group [8]. This finding is similar to ours.

In the present study comprising 381 children, 185 (48.6%) were male children and 196 (51.4%) were female children.

A study conducted by Sangeetha and Priyadarsini on the nutritional status among under-five children in the selected Anganwadi centers in a rural area in and around VMKV Medical College and Hospital, Salem district, Tamil Nadu, has found that among 200 children, 42% were males and 58% were females [9]. This is nearly similar to our study.

In a study done by Naik and Kumar on the nutritional status of Anganwadi children under ICDS in the Rural Field Practice Area of Adichunchanagiri Institute of Medical Sciences have found that the majority of total 770 children - 401 (52.1%) - were males and 369 were females (47.9%) [10]. This is in contrast to our study.

In this study according to the WHO recommended classification, the prevalence of stunting is high, with 243 (63.8%), followed by underweight, 172 (45.2%), and wasting, 79 (20.7%) among the children who attended the Anganwadi centers.

The prevalence of undernutrition among under-five children according to the National Family Health Survey 5 (NFHS 5) in India shows that 29.6% of under-five children were underweight, 31.2% were stunted, and 22.1% were wasted [11]. This is similar to our study

A study carried out by Murarkar et al. on the prevalence and determinants of undernutrition among under-five children residing in urban slums and rural area found that the overall prevalence of stunting is high among children under five, 45.9%, followed by underweight, 35.4%, and wasting, 17.1% [12]. This is similar to our study findings.

A study by Gebre et al. on the prevalence of malnutrition and associated factors among under-five children in pastoral communities of Afar regional state, Northeast Ethiopia revealed that 16.2%, 43.1%, and 24.8% of the under-five children were wasted, stunted, and underweight, respectively, which was found to be very high according to the WHO classification [13]. This is similar to our findings.

In the present study, children in the age group of 36-47 and 48-59 months were found to be more stunted compared to the children of 60-72 months.

These results were found to be in contrast to the findings of the study conducted by Popat et al., India [14], Gebre et al., Ethiopia [13], and Gebreselassie et al. [15] where children of the higher age group were at higher odds of being stunted when compared to the lower age group.

In this study, it was observed that the highest prevalence of morbidity was anemia, 32.80%, followed by upper respiratory tract infection, 20.48%.

A study by Kubde and Kokiwar on the morbidity pattern among children of 0-6 years in ICDS and non-ICDS urban slums of Nagpur city observed that the highest prevalence of morbidity was anemia, 48.1%, followed by respiratory infections, 11.3%, in the ICDS area [16]. This is similar to our study findings.

A study by Phuljhele et al. on the nutritional status and morbidity pattern of children aged 6-60 months and beneficiaries of Anganwadi centers in urban slums area of Raipur city in Central India found that the highest prevalence of morbidity was acute respiratory infections, 26.28%, followed by anemia, 19.83% [17]. This finding is in contrast to our findings.

Limitations

Since the data was collected from the Anganwadi teachers, the family income could not be elicited. So, the socioeconomic status of the family was not included in our study, which could play a vital role in the nutritional status of the children. No appropriate information was available in the record maintained in the Anganwadi centers. The study was confined to Anganwadi children, aged between 3-6 years, which might not reflect the beneficiaries in total.

## Conclusions

The prevalence of stunting was found to be high compared to wasting and underweight among the study children. Nearly 1/3rd of the children had more than one morbid condition. Majority of the children presented with anemia, followed by acute respiratory tract infection. Most of the morbid conditions can be prevented by sensitizing the mothers by Anganwadi workers towards preventive measures through health education. Early and efficient management of common childhood infections needs to be addressed at the level of the Anganwadi.

This helps to break the malnutrition and infection vicious cycle. So ICDS needs to be strengthened to function more efficiently in averting malnutrition among children. Anganwadi workers need to inculcate possible healthy and hygienic practices in the children, which help to improve their health and nutrition. A comprehensive, standardized, continuous, and intensive assessment of the nutritional status of children is recommended.
